# Medical Jurisprudence

**Published:** 1836

**Authors:** 


### REVIEWS AND BIBLIOGRAPHICAL NOTICES.

### Art. IV.—Medical Jurisprudence.

BY A COLLABORATOR.

*Elements of Medical Jurisprudence.* By Theodoric Romeyn Beck, M. D. Professor of the Institutes of Medicine, and Lecturer on Medical Jurisprudence in the College of Physicians and Surgeons in the Western District of the State of New-York, &c. &c; and John B. Beck, M. D. Professor of Materia Medica and Medical Jurisprudence in the College of Physicians and Surgeons, New-York; one of the Physicians to the New-York Hospital, &c. &c. Fifth edition. In two volumes. pp. xx. 661–694. Albany, New-York, 1835.

The task of the reviewer, although not generally deemed so, is yet one of great delicacy and responsibilty, involving not unfrequently the best interests both of individuals and communities. If he is honest and competent, he has it in his power often to benefit alike the author and the public, by bringing into notice works which might, otherwise, be left to decay upon the shelves of the bookseller; but if, on the other hand, he is destitute of these qualifications, he may not only prove ruinous to the former, but, by exciting an improper taste for works that have no merit, or that have a tendency so injurious that they should not be read, he may vitiate the morals of the latter, and thus become a curse instead of a blessing. The reviewer, again, by pursuing an honest course towards his author, may encourage the modest and morbidly sensitive to additional exertion, or he may, by well-timed advice, rebuke the stupid and the insolent, and thus become instrumental, perhaps, in making more elegant and instructive writers, or altogether discard them from a stage which they are not qualified to adorn. Too great leniency, or too much severity, where they are not deserved, are equally improper and unjust, as tending to injure alike the author and the public. There are few men, who, like Byron, are capable of withstanding the severity of a Jeffrey, or who would not shrink, perhaps forever, from the office of authorship, after having been exposed to the shafts of an unjust criticism. Had the noble bard been less talented, less daring, less aspiring, he would probably have bid an everlasting adieu to the muses, and the world might have been thus deprived, if not of one of the most moral, certainly of one of the most vigorous and life-stirring writers of the age; but as it was, the Edinburg Reviewer did no harm—on the contrary, the bearding of the lion-poet only provoked the rage of the animal, and in the "British Bards and Scotch Reviewers," Jeffrey received the full reward of his misapplied temerity.

In no profession, in no avocation of life, should authors be treated with more indulgence than in that of medicine. The reason for this is obvious. As long as our young men are not required to obtain a liberal education, preparatory to their entrance on their medical studies, it cannot be expected that they should make elegant or polished writers, especially if they should take up authorship at an early age; should this be deferred until the meridian of life, and the individual have proper industry, much may be done to correct early deficiencies, and such a person, if he be spared to old age, may leave the world with the reputation of a classical writer. These remarks are not made with a view of discouraging attempts at early writing; on the contrary, we believe that nothing is so well calculated to form a correct style, and to give energy of expression to our thoughts, as exercises of this kind begun in early youth, and constantly practised till old age. All that is designed is to enforce the precept, so often inculcated by the sages of the literary and scientific world, not to *publish* too soon, no matter how much we write. An observance of this precept would be calculated, it appears to us, to make better authors than it is usual to find in our profession, which, instead of being supplied with good productions, creditable to the age in which we live, is but too often inundated with the crudities of the young physician, destitute of solid information, and conveyed in a style that would disgrace the schoolboy. But this is a digression.

The work of Dr. Beck, the title of which we have placed at the head of this article, was first presented to the profession in 1823, and although it remained for many years the only treatise on the subject in this country, yet such was its tardiness to public notice, that a second edition was not called for until last summer. The question will here naturally enough occur to the reader, what was this tardiness owing to? Was it the fault of the work itself, or was it the fault of the profession for whose benefit it was got up? We answer, most unhesitatingly, that the blame was not on the part of the author, or, what is the same thing, on the part of his work, but solely and exclusively on the part of his medical brethren. Those who have anxiously watched the progress of medical jurisprudence in the United States, and are familiar with the frigid indifference with which this science has hitherto been regarded by the majority even of well educated physicians, need no proof to sustain the position which has been here advanced; for it may justly be affirmed, that, had the treatise of Dr. Beck never reached a second edition, the talents and learning displayed in the first, would have been amply sufficient to entitle the author to a distinguished rank amongst the medical writers of the nineteenth century. But to such as have lost sight of the progress of this science—and, unfortunately, the number of them is exceedingly great—it may be well enough to state, that the "Elements" of Beck found their way, at an early period after their publication, into Europe, where they speedily received that attention which ought, in propriety and justice, to have long since been extended to them at home. In England, not less than three editions have been published, enriched with numerous and valuable notes, by two distinguished physicians, Doctors Dunlop and Darwall; and in Germany a translation of it was made some years ago, by a medical gentleman, whose name we do not now recollect. In France, too, the work has been favorably noticed, and, every where, in fact, it has been received as authority on the subject on which it treats.

That the work of Dr. Beck should have received such distinguished notice and patronage from the British profession, whilst it remained comparatively unpatronized and unsought after at home, is doubly singular, when it is remembered that medical jurisprudence was taught as a branch of education for several years in this country, before it received similar favor in Great Britain. To the late Dr. James S. Stringham, Professor of Chemistry in Columbia College, New-York, *is due the honor of having delivered the first course of lectures on legal medicine in this country.* This distinguished physician, from whom Dr. Beck derived his first impulse to the study of a science, which he has since so greatly adorned, was a native of the city of New-York, and a pupil of the celebrated Bard and Hosack. After diligently attending the several branches of medicine then taught in Columbia college, he subsequently repaired to the university of Edinburgh, from which, in 1799, he received the degree of doctor of medicine. Shortly after his return to his native country, Dr. Stringham was appointed to the professorship of chemistry in Columbia college, to which he voluntarily added, in 1804, a course on legal medicine, the popularity of which is said to have been such as to secure its repetition, annually, until his resignation.

In 1813, on the organization of the College of Physicians and Surgeons in the city of New-York, he was selected to fill the chair of Medical Jurisprudence, the first one, we believe, ever instituted in this country. In this situation, however, he was permitted to continue only for a short time; his health, which had become seriously impaired, obliging him to visit the West Indies, where, on the 29th of June, 1817, he went to that "bourne from whence no traveller returns." Dr. Stringham never published any thing on the subject of legal medicine; but we are informed by Dr. Beck, in his preface, that his manuscript lectures have been freely used by him, whenever occasion seemed to indicate the propriety of such a course. As he was the first to bring the subject before the profession in this country, he may be justly styled the "Father of American Medical Jurisprudence;" a title which applies with as much propriety to him as does that of father of American Anatomy to Shippen, or that of American Surgery to Physick.

Traces of the science of Medical Jurisprudence may be found as early as the time of Moses. But it was not until the reign of the emperor Charles V. of Germany, that it was ordained that medical men should be examined in criminal cases. In the celebrated criminal code which was framed under the superintendence of this distinguished monarch, at Ratisbon, in 1532, it is distinctly enjoined that the opinions of physicians shall be formally taken in every case where death has been occasioned by violent means; such, for example, as wounds, poisoning, hanging, and the destruction of the fœtus or infant.

Soon after this period, the science found its way into France and Italy; but it was not until the beginning of the present century that it began to awaken the attention of British physicians, at least to any considerable extent. The first course of lectures on the subject was delivered in the University of Edinburgh, in 1806, by the younger Dr. Duncan, who was succeeded, about seven or eight years ago, by Dr. Christison, the author of the celebrated "Treatise on Poisons." Some idea may be formed of the importance attached to this department of medicine by the British profession of the present day, when it is stated that, in the winter of 1834–5, there were not less than fifty-two individuals engaged in England, Scotland and Ireland, in delivering lectures on it. Of these, twenty-eight are connected with the different universities, colleges, and private medical schools in the city of London; many of them being men of great professional eminence, well known as writers and teachers. To convince the reader of this, it is only necessary to refer to the names of Traill, Thomson, Ramsbotham, Southwood Smith, Davies, Hawkins, Beatty, Ryan, and Blundell; all of them men of great and deserved distinction.

In this country, besides the lectures of Dr. Stringham, commenced in 1804, and consequently two years earlier than those of Mr. Duncan, the Edinburgh professor, Dr. Caldwell, in 1812, delivered a course of instruction on Medical Jurisprudence in Philadelphia, and about three years thereafter, Dr. T. R. Beck, the author of the work under review, was appointed Professor of the same department in the Western Medical College of the state of New-York; a chair which he still fills with distinguished honor. At present, courses of lectures are annually given upon the subject in Boston, New-York, Baltimore, Cincinnati, Lexington and New-Orleans. Strange as it may seem, in the University of Pennsylvania, the oldest, and, until recently, in the talents of its professors and in the number of its students, the foremost medical institution in the country, not one *solitary* lecture has, in all probability, been delivered on legal medicine since the time of the illustrious Rush. Why the trustees and teachers of this venerable school should have lost sight of the utility of this branch of medical science, it is by no means easy to conjecture, more especially when it is remembered that the fees are higher there than in any other college or university in the whole United States. The question may well be asked here, are the officers of this institution, by discarding from her walls one of the most interesting and useful branches of medical science, fulfilling the duties which the medical profession and the country at large have a right to expect from them? Are they keeping pace with the progress of medicine? In a word, are they doing justice to their pupils by giving them an equivalent for their money, and making them expert practitioners, ready to encounter every emergency, both of a public and a private nature? Let those who may feel disposed, answer these queries in such a manner as may be deemed fit; for us to do so, although writing at a distance of more than six hundred miles, it might seem improper. Yet, even at the risk of being harsh and uncourteous, we will venture to express the hope that they will duly reflect upon this subject, and that they will "recollect," to borrow the language of one who, within the walls of their own school, so eloquently inculcated the utility of this science, "the extent of the services which they will be enabled (by teaching this branch of medicine,) to render to individuals and the public; fraud and violence may be detected and punished; unmerited infamy and death may be prevented; the widow and the orphan may be saved from ruin; virgin purity and innocence may be vindicated; conjugal harmony and happiness may be restored; unjust and oppressive demands upon the services of their fellow citizens may be obviated; and the sources of public misery in epidemic diseases may be removed."*

Jefferson College, scarcely out of her teens, yet bidding fair soon to outstrip, if she has not already done it, her older sister, had formerly a professorship of Medical Jurisprudence, occupied by the late Benjamin Rush Rhees, M. D. For five or six years, this gentleman lectured on this science, in connexion with the Institutes of Medicine, not only with much credit to himself, but with much advantage to his pupils, and we well remember the interest with which we used to listen to his instruction, conveyed in a style at once eloquent and impressive. On the death of Professor Rhees, which unfortunately occurred too soon for the cause of the science which he so ardently cultivated, the chair was abolished; and since that time Jefferson College may be considered as fully entitled to a share, at least, of the censure that ought to rest on every medical school in which this science is neglected. We trust, however, that our young alma mater will soon perceive the error of her ways, and that, being conscious of it, she will set about and amend them.

* Rush. Introductory Lectures, p. 392.

It is thus, by short but regular strides, that the science under consideration has attained its present elevated and dignified position; and although scarcely thirty years have elapsed since it became first known, even by name, in this country and in Great Britain, the amount of productions in this department has reached many thousand volumes, besides almost innumerable papers in journals and scientific collections. In Wilberg's Bibliography of Medical Jurisprudence, which extends to the year 1819, mention is made of no less than 2980 separate works in forensic medicine, in the different languages of Europe. Now, if we average these at no less than two volumes each, which no doubt falls short of the truth, and add the probable number of works that have been published since that period, the amount will be little short of eight or ten thousand volumes.

The first treatise on this subject, in any language, probably, was composed in 1598, by an Italian physican of the name of Fortunatus Fidelis; who was followed soon after by the celebrated Zacchias, to whom, in consideration of his great work, has been usually awarded the title of the "Father of Juridical Medicine." In the German language, the most important works are those of Bohn, Teichmeir, Hibenstreit, Plenck, Schlegel and Metzgér, amongst the older writers, and those of Bernt, Henke, Emmert, Gmelin, Wagner, Remer, Hecker, Hoffbauer and Jæger, amongst the more recent. In France, besides the names of Mahon, Fodere, and Orfila, which have long since become familiar to every tyro in medicine, may be enumerated those of Marc, Ballard, Capuron, Briand, Biessy, Esquirol, Falret, and Georget. In England, the most distinguished writers are Male, Smith, Paris and Fonblanque, Beatty, Arrowsmith, Ryan and Chitty. The works of the two latter have been republished in this country, and as they have both been for some time before the profession, I shall only observe, concerning the former, that, although in many respects a useful compilation, it is entirely too compendious for the more advanced student; and respecting the latter, that Mr. Chitty, however well he may be qualified, by his talents and industry—and he displays a goodly share of both—to instruct his brethren of the Inner Temple, he is by no means calculated to enlighten the members of the medical profession, of whose "mysteries profound," he has little or no knowledge. These works, together with the admirable one of Dr. Beck, and the Catechism of Dr. Williams, of Maine, a performance too contemptible to be criticised, constitute all, with the exception of some detached papers, that has thus far been published on legal medicine in this country, and which will, for a long period to come, preclude the necessity for any thing more in this department of medical science.

Having thus presented a rapid sketch of the history of medical jurisprudence, for some of the materials of which we are indebted to the Introductory Discourse of the work before us, we proceed, secondly, to offer an analysis of such portions of the "Elements" as may seem most interesting and instructive to our readers. The first volume, to which we shall more particularly direct our attention on the present occasion, comprehends thirteen chapters, including the subject of feigned and disqualifying diseases, impotence and sterility, doubtful sex, rape, pregnancy, delivery, infanticide, legitimacy, presumption of survivorship, age and identity, insurance on lives, and mental alienation. Passing by the first two chapters as possessing *comparatively* little interest to the generality of physicians, we come to that on impotence and sterility, running through an extent of twenty-six closely printed pages.

"IMPOTENCE AND STERILITY.

"A knowledge of this subject may become necessary in various ways, before judicial tribunals. An individual accused of committing rape, has been known to plead that he was physically incapacitated; while the legitimacy of children has been contested on a similar plea. These examples are sufficient to show the necessity of a brief notice of the physical signs of impotence, even were they not connected with the subject of divorce."—*p.* 67.

The laws of Moses allowed divorce at the pleasure of either party, and this is the principle which still prevails amongst many of the uncivilized nations of the present day. The Christian law, however, declares marriage to be indissoluble; and hence the canon law, which was founded on this precept, permitted divorce only where the defect was known to have existed prior to the wedlock. Justinian, who was the first monarch that prescribed the mode of obtaining divorce, ordained that if the imbecility continued for two years after marriage, the injured party should be entitled to redress. The law of England on this subject is, that a dissolution of the marriage contract may be granted, whenever it is proved that corporeal imbecility existed before the celebration of the nuptial rites. Imbecility, however, arising after marriage, is not a sufficient ground for divorce, as there was no fraud in the original contract. The French code does not expressly declare that absolute and incurable impotence is a dissolving cause of marriage; yet such would seem to be the inference from the course of proceedings under it. In the United States generally, the English law has been adopted on this subject: thus in Pennsylvania it is enacted by the act of the 13th of March, 1815, "That if either party, at the time of the contract, was and still is naturally impotent, or incapable of procreation, it shall and may be lawful for the innocent and injured person to obtain a divorce." In our own state, the applications for a dissolution of the marriage contract are every year exceedingly numerous, and there is much reason to believe that divorce is often granted for the most trivial causes. In the state of New-York, the laws are nearly similar to those in Pennsylvania; wisely ordaining that impotence supervening after marriage, is not a sufficient cause for separation.

"The causes of impotence have been variously divided by different writers; but I conceive that I shall be best enabled to give a comprehensive view of them, by adopting the arrangement of Fodere, into *absolute, curable* and *accidental or temporary*."—*p.* 70.

The absolute causes of impotence in the male, or those for which there is no relief, consist chiefly in the want or absence of the testicles and penis, a vicious direction, or great or imperfect development of this organ, and a preternatural course or opening of the uretha.

Formerly the testicles were not deemed essential to procreation. Aristotle was led to such a conclusion from having observed that a bull was capable of impregnating the cow after castration; a fact which depended, no doubt, upon the quantity of semen retained in the spermatic reservoirs, conferring fertility upon an intercourse which took place immediately after the operation. This question was, at one time, much agitated by some of the German schools, but with what results it does not appear. The subject, it seems to us, can only be determined by experiments on animals; but in the meantime it may be observed that probably few persons, if any, could, after undergoing so severe an operation, have a prolific intercourse, as the pain would, in all likelihood, be so great as to prevent erection; besides, the fluid remaining in the seminal vesicles would, in the course of a few days or weeks, or at all events before the wound could be healed, be absorbed into the system, or be rendered so weak as to lose its fecundating properties. The most, then, that a person thus circumstanced could possibly do, would be to have one or two connexions, and no more.

A highly interesting case, bearing directly on the question before us, is mentioned by the author, and which we will endeavor to condense for the benefit of the reader. It came under the observation of Sir Astley Cooper, who states that he performed the operation of castration for the second time upon the same individual, in 1801, for chronic abscess of the testicle; and that on visiting the patient four days subsequently, he informed him that he had, during the preceding night, a seminal emission. The person had been married prior to the first operation, and for nearly twelve months after the complete removal of his testes, he declared to Sir Astley that he not only had erections, so as to enable him to have sexual intercourse, but actual emissions, or at least sensations of that nature. After this, he was able, at distant intervals, to command erections, but experienced no longer any thing like seminal evacuations, either as regards feeling or in point of fact. No mention is made whether any issue was the result of these connexions; an omission much to be regretted, when we consider the interesting bearing which the case posesses in relation to legal medicine. In all likelihood, however, there was no offspring; and it appears very clear to our own mind, that the emission which occurred four days after the last operation, was the last one of a truly seminal nature; the others, no doubt, were merely *prostatic.*

The absence of the testes from the scrotum does not necessarily imply impotence. These organs, it is well known, are formed originally in the cavity of the abdomen, from whence they *ascend*—for it cannot, in the great majority of cases, be a descent, as stated in the books—into the scrotum between the seventh and ninth months of utero-gestation, and occasionally, indeed, not until some time after birth. When they remain in the abdomen during life, it does not necessarily follow that the individual thus circumstanced, must be impotent. Fodere, a celebrated French jurist, in fact, asserts, that persons of this description, instead of being disqualified, are not unfrequently remarkable for their prolific powers and sexual vigor.

The testicles may be destroyed by weapons, or they may be extirpated on account of disease, or they may be removed wilfully, waste from organic disorder, or be absent as a congenital defect. Baron Larrey states, that during the French campaign in Egypt, many of the soldiers attached to Bonaparte's army, were attacked with a wasting of the testes. These organs lost their natural sensibility, became soft, and gradually shrunk until they were finally not larger than a small bean. No venereal disease, it would seem, preceded or accompanied these attacks, and hence Larrey was induced to attribute this atrophy entirely to the use of a species of brandy, made of dates, in which the soldiers more or less indulged. When both testes were thus wasted, the individual invariably became impotent, he lost his flesh, became depressed in spirits, and grew weak in intellect. Similar cases have been reported by Fodere and other writers. From the intimate connexion between these organs and the cerebellum, the seat, according to the phrenologists, of the appetites and passions, severe blows inflicted on the back part of the head, or fractures in this region, may give rise to impotence; though the reverse is sometimes the case, the patient being occasionally extremely salacious. In a case reported by my friend Dr. Donne, of Louisville, Kentucky, in which the cerebellum was wounded by a musket-ball, the individual labored under constant priapism until the very moment he expired.

Malformation of the epididymis sometimes induces incurable impotence. The same is the case with the vas deferens, which sometimes opens in a cul-de-sac, as in the instance referred to by the author; at other times this canal may be obstructed by cheesy, calcareous or curd-like matter, or its walls may be united by adhesive inflammation, and thus prevent the escape of the seminal fluid. In the body of a stout muscular subject, dissected by professor Mayo, of London, the vas deferens, instead of ascending in the spermatic cord in the usual way, was reflected downwards, and terminated at the rete testis. In place of the seminal vesicles, on one side there was a narrow slip of dense, fleshy substance, perfectly void of an interior cavity, whilst on the other every vestige of this reservoir was wanting. The structure of both testicles, however, was natural. (Outlines of Human Physiology, p. 363). In all such instances, a post-obit examination could, of course, alone reveal the real nature of the defect.

Obliquity, tortuosity or bifurcation of the penis, may be considered as so many causes either of temporary or permanent impotence. If the organ is so much bent out of its natural course as to prevent the semen from entering the vagina, there can be no doubt as to the disqualification being complete. The same consequences may arise, as is very properly observed by the author, "from an amputation of the virile organ, a scirrhous or paralytic state, induced by injury to the nerves or muscles of the part, and an unnatural perforation of the penis, or, in other words, the extremity of the canal of the urethra, terminating at some other than its natural situation. When this happens on the upper part, it is styled *epispadias*, when below, *hypospadias*." This malformation, is not, however, in all, or even perhaps in the majority of cases, an absolute cause of impotence, many instances being cited by professor Beck in which it was remedied by a surgical operation, or in which, notwithstanding its presence, the individuals had a numerous offspring. What is more remarkable than all, is that this deformity may sometimes be transmitted from father to son. Dr. Frank, of Germany, has seen a case transmitted through three generations. When the opening of the canal takes place in the perinæum, or above the pubes, as it occasionally does in monsters, the individual will necessarily be impotent.

Ossification or a cartilaginous condition of the septum of the penis, may become a cause of temporary or incurable impotence, by preventing copulation. A case of this kind once occurred to Dr. McClellan, of Philadelphia. The individual, already considerably advanced in life, but with sexual powers unusually vigorous, was unable to cohabit in consequence of the virile organ being so much arched towards the perinæum, as to render it impracticable to introduce it into the vagina. On making an incision along the dorsal surface of the penis, the pectiniform septum was found to be converted into a plate of cartilage, the removal of which was soon followed by a complete restoration of the functions of the organ.

Other causes may induce this affection, such as strictures of the urethra, phymosis, peraphymosis, scirrhous enlargement of the neck of the bladder, or prostate gland, or whatever may impede the exit of the semen from its reservoirs. To these may be added scrotal hernia, hydrocele, and various diseases of the spermatic cord. Many of these causes, however, it is obvious, may be successfully removed by surgical treatment.

Occasionally men have been known to labor under *moral* impotence, caused by excessive emotion, surprise, great modesty, disgust, chagrin, jealousy, and various other circumstances to which it is not necessary to refer. "There are no facts," says Dr. Ryan, "which so clearly prove the influence of the moral over the physical state of man, as the phenomena of erection. A lascivious idea will arise in the mind amidst our gravest meditations, the virile organ will answer its appeal, and will become erected, and fit for the functions which nature has confided to it; but another thought arising, will instantaneously extinguish, with the most frigid indifference, all our amorous transports." A most remarkable instance of moral impotence came under our own notice during the past summer. A gentleman, about 35 years of age, put himself under our care on account of a general debility of the genital organs, attended with frequent nocturnal pollutions, and a difficulty, at times, of commanding erection. He had formerly indulged in the detestable vice of onanism, but at the period we first saw him, his health did not seem to be materially impaired. After correcting the secretions of the liver, and of the alimentary canal, he was directed to take mineral tonics, to bathe the perinæum twice a day in cold water, and to take gentle exercise in the open air. By persisting in this plan of treatment, the sexual organs evidently became more vigorous, and as he was engaged to be married, we felt anxious, as the best means, as we thought, of effecting a speedy cure, that he should anticipate his engagement. He did so, and was accordingly married several months earlier than he had at first intended. Contrary to our expectations, however, and much to his own annoyance, all his attempts at sexual intercourse proved abortive; for although he could command an erection, and would even have occasional seminal emissions whilst lying with his wife, yet he found as soon as he approached her, that the virile organ became perfectly flabby, and so continued in spite of all his efforts. In this condition he again requested our advice, when, in consultation with our learned and respected colleague in the chair of Theory and Practice, he was put upon the use of phosphorus, in the dose of one-twelfth of a grain three times daily. He persevered in the employment of this potent remedy for about a fortnight, but the only effects which he experienced from it were an increase of his nocturnal pollutions, and more frequent erections. Becoming discouraged, he at length abandoned every thing like medicine, determined to see what time would accomplish in a case of such unusual obstinacy. In December last, however, he applied to us again, and we now prescribed a mild decoction of St. John's wort, assuring him that this medicine had done wonders in the hands of the celebrated John Hunter, and other eminent physicians, and that there was no doubt whatever but that it would prove completely successful in his own case. He left our office in the "full flush of hope," went home and tried the medicine which had been suggested to him, and when he met us again a few weeks after, he seemed to be one of the happiest beings in the world; thanking heaven that all his difficulties had disappeared, and assuring us, to use his own language, that he could do the thing as well now as any body.

This case, which from its unusual protractedness, embracing a period of nearly six months, is one of great interest in a medico-legal point of view, was unquestionably one of moral impotence, produced by the extraordinary modesty of the individual, and by an over-anxiety about the result of his sexual efforts. To the same class of cases, but in which impotence was no doubt the result of disgust, may be referred that of Lord Rochester, which excited so much interest in the reign of James the First. This is the more probable, as it was shown on the trial that his lordship, although unable to effect carnal intercourse with his wife, had been able to deal with other women, both before and after his marriage.

An individual occasionally dies from protracted disease, and eight or nine months after, the widow is delivered of a healthy and full-grown child. When a case of this kind occurs, it is almost sure to lead to a legal prosecution, especially if there is no other issue from the marriage. We find accordingly that the subject has not been overlooked by the author.

"The diseases that are considered compatible with connexion, are those which do not affect the head and sensitive system primarily, and are not accompanied with great debility. Inflammatory and catarrhal fever are of this class. So also in asthma and the early stages of phthisis pulmonalis, the power is preserved.* Some diseases appear to stimulate the generative organs; and others, although accompanied with pain, are said to excite desire. Of the first, may be named a calculus in the kidneys or bladder; and to the last belong gout and rheumatism.†

A man named Aurelius Lingius, aged sixty years, had been affected, during the last two years of his life, with occasional attacks of fever, accompanied with gouty pains, which at intervals, made him extremely ill. For the space of two months, however, he appeared on the recovery; when, being seized with a fever and ague, he died. His wife declared herself pregnant, and six months after his death, was delivered of a healthy child. Its legitimacy was contested, on the ground that the husband, before his last illness, had been incapable; and this opinion was corroborated by his own confession to the physician attending him. His wife allowed the truth of this statement, but asserted that his powers had returned some time before his decease. In this state of the case, Zacchias was consulted; and he decided in favor of the chastity of the wife, for the following reasons: Aurelius had been twice married, and by each wife has had several children. The disease under which he labored was a heating one, and his powers were probably perfect during the period of convalescence. His age does not prevent the possibility of his producing pregnancy in the female. Symptoms of this were present during his life-time; and although he was known to be extremely jealous, yet his affection remained undiminished towards her. And finally, the intervals of ease that accompany articular pains, together with the fact that she always reposed in the same bed with him, were, in the mind of Zacchias, conclusive arguments. The judges decided in favor of the female.‡

In connection with the facts already stated, it may be proper to add a circumstance suggested by the author just quoted. He deems it possible that certain diseases may so change the state of the system, as to produce an alteration in the generative power. He quotes the testimony of Avenzoar, who had no children during the whole period of youth, but became a father shortly after recovering from a violent fever. And also the case which came under his own observation, of an artificer, who lived twenty-four years with his wife without issue: shortly after his convalescence from illness, he became a father, and afterwards had many children.§

The diseases which we may rationally suppose will prevent cohabitation, are the following: A mutilation, or severe wounds of the sexual organs--carcinoma of the testicles or penis—gangrene of the lower extremities—immoderate evacuations of blood or bile, or of the

---

* Orfila's Lecons, vol. 1, p. 136. Louis, a late writer on consumption, denies the truth of this opinion, so far as to limit it only to the earliest periods. In more advanced stages, he is convinced that it decreases with declining strength. The Editor of the London Medical Repository (vol. 25, p. 106,) remarks on this: "We have no doubt, that in some examples of phthisis, both the power and the propensity to gratify it, have existed up to the very day of the patient's death."

† A friend of mine, who studied in the hospital of New-York, informed me, that after recovering from the yellow fever, the patients displayed most furious sexual passion, to the great inconvenience of the nurses and other female attendants."—Dunlop.

‡ Zacchias, Quest. Med. Leg. Consilium, 23.

§ Zacchias, vol. 1, p. 171.

fæces—scorbutic cachexia—marasmus—peripneumony and hydrothorax—anasarca in its perfect state, particularly if accompanied with an infiltration into the sexual organs—nervous and malignant fevers, particularly if they affect the brain, and are accompanied with great debility and loss of memory—all affections of the head and spinal marrow, whether from a fall, blow, wound or poison;* or from internal causes, as apoplexy, palsy, or other comatose diseases. If the infant is conceived whilst the husband has been known to have labored under either of these maladies, the presumption is certainly against its legitimacy. So also, if he be afflicted with leprosy, venereal ozœna, severe cutaneous diseases, or insanity, we may reasonably doubt the fact of cohabitation, from the fear that we may suppose the female has experienced, lest she should be contaminated, or from the dread that she has entertained of having communication with the individual."—*p.* 79.

We now come to the consideration of impotence in the female, which, like that in the male, may be divided into curable and incurable. The latter, as stated by the author, may arise, first, from an obliteration or thickening of the sexual organs, so as to prevent any introduction of the virile member; secondly, the total absence, or extreme brevity of the vagina; thirdly, a fistulous communication between this tube and the bladder or rectum; fourthly, irreducible prolapsus or retroversion of the uterus; and fifthly, a carcinomatous condition of this organ or the vagina, inducing severe pain during the venereal congress. Under the first head of incurable causes, an interesting case is cited from the second volume of the New-York Medical and Physical Journal, where it was originally reported by Dr. Mott. The female was twenty-three years of age, during the last two of which she had been married. Her health was extremely good, but she had never seen the least indication of the menses. About every lunar month, she experienced some slight uneasiness about the pelvis, followed for a day or two by active diarrhœa. The external parts were well formed, but no vagina could be discovered, although Dr. Mott introduced the knife between two and three inches high. Her husband could never effect any intercourse; and from the fact that she has never experienced the least

* Fodere mentions the case of a person, aged forty, who labored under temporary impotence during the space of six months, from exposure to charcoal vapors. This state of the system was left after the recovery from the immediate danger. Vol. 1, p. 382.

sexual desire, Dr. Mott was induced to believe that both vagina and uterus are wanting. Cases of a similar nature are mentioned by Dr. Davis, in his Obstetric Medicine, by Dr. Lee, in the London Cyclopædia of Practical Medicine, by the author of the article "Cas rares," in the Dictionnaire des Sciences Medicales, and in fact, in almost every medical journal of the present day.

The curable causes of impotence are, a dense substance closing the orifice of the vagina; extreme narrowness of this tube, occurring either as a natural condition, or as the effect of accident or morbid growths, such as tumors and callosities, cicatrices remaining after the cure of ulcers, or from lacerations after difficult labor; imperforation of the mouth of the uterus; long continued hemorrhage; recent prolapsion of the uterus or vagina; and even protracted fluor albus. All these causes prevent connexion from the pain that occurs, or the diseased state that is present.

Sterility should be considered as existing exclusively in the female, inasmuch as the generative process on the part of the male is altogether confined to the act of intercourse, and if this be properly performed, no further question can be made as to the influence derived from him. The causes of sterility or barrenness are of two kinds, constitutional and local. The effects of the first can be known only by the event of unfruitfulness in the married state; they may endure for many years, and then recede, the woman in the meantime remaining perfectly healthy, with desire and enjoyment of connubial intercourse. On the other hand, a female may bear one or two children soon after she is married, after which she may cease to be fruitful until she is considerably, sometimes very far, advanced in life, when the powers of conception returning, she may have a numerous offspring. Instances of this description might be cited in great numbers, but one of the most remarkable with which we are acquainted, occurred during our residence in Philadelphia. A highly respectable lady, the wife of a captain in the merchant service, and an intimate friend of the writer of this article, had been married twenty-seven years without offspring; at the end of this period she conceived, and in due time was delivered of a fine healthy boy. Zacchias refers to a case of twenty-four years standing. In both instances, there was only one child.

"The causes of sterility, of an incurable nature, and sensible to the sight or touch during life, may be stated thus: A schirrous or cartilaginous uterus; stricture in the cavity of that organ; a polypus in the interior of the uterus; enlarged and schirrous ovaria. The want of the uterus, should that occur, is seldom positively known till after death. The causes which may be curable, are, obliquity in the position of the uterus; too great irritability of that organ; excessive menstruation; leucorrhea; retention of the menses. This last, however, is not by any means a certain cause of sterility, as women have become pregnant without the menses ever occurring."—*p. 87.*

### DOUBTFUL SEX.

We perceive nothing in this chapter, taken almost *verbally* from the article on Hermaphrodism in Dr. Brewster's Edinburg Encyclopædia, to detain us. The only thing new in this part of the work, is a brief analysis of the recent researches of St. Hilaire, of Paris, on Hermaphrodism. This writer divides the genital apparatus into six segments, three on each side, which, in several respects, are independent of each other. The first two segments include the testicles and ovaries; the next, the seminal vesicles, and the womb or prostate; and the last two, the penis and scrotum, the clitoris and vulva.

"When the number of these parts is not changed, and there is simply a modification in their developement, we have the FIRST CLASS, or *hermaphrodism without excess.* This again is subdivided into four orders: 1. *Male hermaphrodism,* when the generative apparatus, essentially male, presents in some one portion the form of a female organ—as a scrotal fissure, resembling in some respects a vulva. 2. *Female H.* where the apparatus, though essentially female, yet offers in some one portion the form of a male organ, as in the excessive development of the clitoris. 3. *Neutral H.* when the portions of the sexual apparatus are so mixed up, and so ambiguous, that it is impossible to ascertain to what sex the individual belongs. 4. *Mixed H.* when the organs of the two sexes are actually united and mixed in the same individual. Of this there are several species: *Alternate,* when the deep organs belong to one sex, and the middle to the other, while the external present a mixture of both. *Lateral.* In this, the deep and middle organs, when viewed on one side of the median line, appear to belong to the male sex, while on the other they are female; the external organs, as in the former species, are partly male and partly female. *Hemilateral.* *Interchanging.*

The SECOND CLASS includes all anomalies with excess of parts, and is divided into three orders: *Complex Male H.* where we find, with an apparatus essentially male, some supernumerary female organ, as a uterus, &c. 2. *Complex Female H.* with the addition of a male organ, as a testicle, &c. to an apparatus essential female. 3. *Bisexual H.* where a male and female apparatus exist in the same individual. M. St. Hilaire allows, however, unequivocally, that the external organs (as a penis and clitoris) have never been found perfectly double. 'The researches of modern anatomists have completely set at rest the long debated question of hermaphrodism, in the vulgar acceptation of the word. It is anatomically and physiologically impossible.' "—*p.* 108.

Towards the close of utero-gestation, the genital organs constitute the most marked distinction between the sexes; but if these organs be examined at an early period of embryotic life, it will be found that there exists so close an analogy in their general conformation, that we must at once conclude that both are formed according to one common type. The testicles and the ovaries have a striking similitude; they are nearly of the same form and size, are both situated originally at the side of the lower part of the abdomen, are made up nearly of the same organic elements, and, finally, they perform precisely the same functions, both being essential to procreation. The uterus and the seminal vesicles bear a great resemblance to each other, both as regards their position between the bladder and the rectum, and their mode of connexion with the Fallopian tubes and the deferential ducts, the former serving as excretory canals to the ovaries, the latter to the testicles. The clitoris, again, may be likened to the penis, which, in the early months of fœtal life, it generally so closely resembles as to render it difficult to point out the distinction; whilst the labia may be compared to the scrotum, which is occasionally cleft, so as to give rise to an ambiguous state of the genital apparatus.

### RAPE AND DEFLORATION.

These topics are very ably treated by Dr. Beck, the chapter in the which they are discussed, being decidedly one of the most interesting and elaborate in the whole book. "Rape is the *carnal knowledge* of a female, forcibly and against her will;" whereas defloration may be defined to be that species of sexual intercourse, in which the female, giving her consent to the act, receives no bodily violence. Authors, both medical and legal, differ considerably as to what should constitute the crime of rape or defloration; or, in other words, as to the precise meaning which should be attached to the phrase "carnal knowledge of a female;" some contending that mere penetration is sufficient, whilst others allege that there should be both penetration and emission of the seminal fluid. Considering the revolting nature of the offence, it might reasonably be presumed that the former of these circumstances alone was sufficient; for the fact simply of the emission, superadded to the penetration, could not possibly, it seems to us, enhance its enormity. If, indeed, it be taken for granted that both penetration and emission are requisite to constitute the perfect accomplishment of the crime, it must necessarily follow that the culprit will often escape punishment, and thus frustrate the ends of justice. A young female, in the struggles which she might make for the preservation of her chastity, and in the almost utter unconsciousness which might result from these exertions, might be unable to afford satisfactory information in regard to the emission, or she might be so young or inexperienced as to be ignorant of what constitutes an emission; yet would it be right, because she could not afford the requisite evidence on this point, that she should be deprived of the benefits of a legal prosecution? As well might it be required of a man, shot by another, that he should have seen the ball of his adversary, in order to enable him to obtain redress for the injury; the case would not be a bit more absurd; and yet if such a person should show his wound in court, or testify to its present or former existence, he would at once be believed, although he might never have seen the ball or weapon by which it was inflicted.

The views of the author, in relation to this subject, so perfectly coincide with our own, that we cannot resist the pleasure of quoting his own language. In speaking of the possibility of committing this crime, and of the cases in which it may be consummated, he makes the following pertinent remarks:

"The necessity of establishing the fact of emission must prove an insuperable barrier to any conviction. We may divide females, with reference to this subject, into two classes—the young, unmarried persons; and the married, or those accustomed to sexual intercourse. As to the first, it may be considered very improbable that they could be conscious of this, while laboring under the influence of terror, severe pain, faintness or insensibility. And to this class also, belong those of a very tender age, who are totally ignorant of the nature of the crime, and of what is necessary to complete it.

"It is, however, urged, that there is great propriety in requiring this testimony from married females; and that if they are not sensible of that 'which constitutes the very essence and climax of feeling,' their declarations do not deserve much credit as to the other parts, in which a less degree of poignancy of sensation is requisite. I confess, that language of this kind appears to me misapplied. If proper resistance be made, where the contest is solely between two individuals of strength in any degree proportionate, the crime can scarcely be completed without violent personal injury to the female. The exhaustion that must be present, superadded to mental agitation, leave us some reason to doubt whether this enjoyment can be realized. And it also deserves consideration, that if the resistance has been *complete throughout*, such pain may ensue from the repeated attempts to effect the crime, as to dull all sensation on this point."—*p.* 142.

These views are in the highest degree just and philosophical, and if they were fully comprehended and rightly appreciated, they would soon lead to the removal—the total abolition—of that absurd and injurious distinction which has been so much insisted on by certain legal and medical writers. In the case of the virtuous female, it would matter but little whether the penetration was attended with or without an emission, as the circumstance, if known, will equally consign her to misery and disgrace.

The punishment for this offence has varied in different ages and in different countries. By the Mosaic laws it was punished with death; and the same was the case with the Athenian. The Roman laws, likewise, regarded it as a capital offence, in addition to which they enjoined the confiscation of the effects of the criminal. Charlemagne punished with death whosoever violated the daughter of his master. In the early part of the reign of Edward III, it was considered merely as a trespass, and therefore punished simply by fine and imprisonment. This lenity, however, was soon found to be productive of the most terrible consequences, on which account it was shortly afterwards made felony, without benefit of clergy. This law, enacted in the time of Queen Elizabeth, is, we believe, still in force in England. In France, the punishment seems to be more mild, not at all proportioned to the nature of the offence, the only penalty being hard labor for a limited number of years. In our own country, the punishment varies in the different states. In South Carolina, Massachusetts, Delaware and Rhode-Island, the penalty is death; whereas in Ohio, Illinois, Indiana, Pennsylvania, New Jersey, and most of the other states, confinement in the penitentiary for a term of years, or for life, is prescribed.

The *physical signs* of virginity rest, in great measure, upon the state of the genital organs, such as their size, the presence or absence of the hymen, the narrowness or rigidity of the vagina, and the firmness of the labial frænum.

There is, perhaps, no topic connected with anatomy and forensic medicine, which has given rise to greater inquiry, or led to more acrimonious discussion, than the existence of the hymen. This structure, situated at the infero-lateral part of the orifice of the vagina, is a thin, delicate membrane, consisting of a duplicature of the mucous lining of the vulva. Dr. Beck, as well as most other writers, describes it as being of a semilunar or circular form, but in the great majority of cases that have come under our own notice, we have found it to be of an oblong, oval shape, with a rounded central aperture. In general, it is thin, weak and easily torn; more rarely, it is dense, firm, resisting, and arranged so as to form a complete valve, which effectually prevents the discharge of the catamenial flux.

Amongst those who have admitted the existence of this membrane, the author has enumerated the names of Fabricius, Albinus, Ruysch, Morgagni, Haller, Diemerbroek, Heister, Riolan, Sabatier, Cuvier, Blumenbach, Denman, Dewees, Godman, Gavard, Senn. The latter of these writers, who was for a number of years physician to the "Maternity Hospital"

at Paris, indeed asserts that he did not fail, *in a single instance*, to find the hymen in an examination of upwards of three hundred children, from two to four years of age; and the testimony of Gavard is nearly to the same effect. "Cuvier," our author states, "has not only found it in females, but also in mammiferous animals; and thus gives strong evidence of its existence by analogy." Here we must beg leave to disagree with Professor Beck. Cuvier, it is true, describes a small fold which he considers as analagous to the human hymen, in some monkeys, the mare, ass, dog, cat, and otter; but it appears very clearly from his own account, that the structure in the above named animals bears only a very remote resemblance to the hymen of the human female; in other words, there is no hymen at all.

Those, on the other hand, who have denied the existence of this "chastity valve," or considered it as a preternatural or morbid occurrence, are Ambrose Pare, Palfyn, Pinæus, Columbus, Dionis, Buffon, and Dr. Chapman, the distinguished professor of the Theory and Practice of Medicine in the University of Pennsylvania. The weight of evidence is thus decidedly in favor of the existence of this membrane; and we are clearly of the opinion, both from our own observations and those of others, that it originally exists in every female infant; but owing to the great ease with which it may be destroyed by mechanical violence or disease, it is seldom found in the adult woman. Of this, however, we have seen no less than two cases; the one occurred in a negress, about twenty years of age, whose dissection we witnessed, eight or nine years ago, in the University of Pennsylvania; and the other, in an idiot girl, 17 years of age, who died at the Cincinnati Hospital in the winter of 1834. In both the membrane was perfect.

By the Jewish legislators, the presence of the hymen was universally regarded as a certain sign of virginity, and the opinion, unfortunately, has been countenanced by some distinguished writers of a more recent period. The following circumstances, according to Dr. Beck, require to be noted before we form an opinion concerning this membrane as a sign of virginity.

"*It may be wanting from original mal-conformation, or it may be destroyed by disease or some other cause, and yet the female be pure.* Thus the first menstrual flux, if the aperture be small, may destroy it —or an accident, as a fall—or disease, as for example, an ulcer, may totally obliterate it. There have certainly occurred instances, where the pressure of the confined menstrual fluid has produced its destruction. Again, in the place of the hymen, are sometimes found the *carunculæ myrtiforme*. Tolberg, according to Fodere, and also Belloc, have made this observation on dissection. They were, however, round, and without a cicatrix, and in this respect very distinct from the organs usually so termed. *This membrane may, on the other hand, be present, and yet the female be unchaste; nay, she may become pregnant without having it destroyed.* Zacchias remarks, that it will not be ruptured when it is thick and hard. A disproportion between the organs, or connexion during the presence of menstruation, or fluor albus, are also mentioned by him. Gavard, whom I have already mentioned, found it perfect in a female thirteen years of age, who was laboring under the venereal

"In those cases where this membrane is found thickened, an operation has often been necessary. Pare relates of a mother who applied to him to examine it; and on dividing it, it was seen to be of the thickness of parchment. A similar case happened to Ruysch, of a female during labor, in whom he had not only to divide the hymen, but also another non-natural membrane placed farther back. Immediately after the operation, the child was born. Baudelocque, Mauriceau, Denman, and other writers on midwifery, adduce many instances illustrating the same fact.

"These observations certainly lead us to doubt whether the presence or absence of the hymen deserves much attention; and I believe the opinion of physioligists generally is, that it is an extremely equivocal sign. I am, however, unwilling to go as far as most of the later writers on legal medicine, who virtually reject it altogether. While it must be allowed that it can very often be destroyed by causes which do not impair the chastity of the female, we are justified, I think, in attaching considerable importance to its presence. It would be difficult to support an accusation of a rape, where the hymen is found entire. I feel therefore justified in retaining it among the signs of virginity, although it should always be considered in connexion with other physical proofs."—*p.* 113.

This is, perhaps, the limit beyond which it would be imprudent to go. In all cases, particular inquiry should be made as to the reputation of the accuser; if her character has been uniformly good, her "walk and conversation" perfectly chaste, and, finally, if there are marks of a lacerated state of the hymen; it may be concluded that her accusation is not without proper foundation; whereas, if all the contrary circumstances exist, there will be just ground for pronouncing a contrary opinion.

These remarks apply with equal force to the *myrtiform caruncles;* these bodies, of which there are two or three on either side, are generally supposed to result from the rupture of the hymen; but we are informed by Hamilton, Beclard, Conquest, Blundell, Hildebrandt, and others, that they are nothing but folds of the mucous membrane, and that they exist frequently where the hymen is present. On the other hand, professor Orfila asserts, that in several hundred dissections made by him of children from two to fourteen years of age, and in whom, of course, the hymen was present, he could not detect the existence of the myrtiform caruncles. Velpeau has endeavored to account for these discrepancies, by what he deems his own discovery. Four carcuncles, he observes, are commonly seen at the mouth of the vagina, corresponding to the four extremities of the respective diameters of this opening. Now two of these belong to the middle columns of the vagina, that namely which is near the urinary meatus, and that which is near the fourchette, whereas the other two only are the remains of the hymen. They may thus, he supposes, co-exist. These last he calls the *lateral caruncles*, in contradistinction to the other, which may be termed the *central caruncles*. This *discovery*, as the Frenchman styles it, has not yet been verified by other observers, and hence, in the meantime, it must be taken for what it is worth.

Where a rape has been actually committed, the genital organs, especially in young girls, will, generally, be more or less tumefied, contused, red, and sometimes slightly ecchymosed. If the hymen was present, it will be found lacerated, and covered with blood, or sero-sanguinolent fluid; the clitoris will also be swelled, and all the parts will be hot, and more or less painful. These phenomena rarely remain distinct beyond three or four days, and they may be said always to disappear much sooner in females who have been accustomed to sexual intercourse, or who are laboring at the time under leucorrhæa or some other vaginal discharge, than in the young and unmarried. Hence, as observed by the author, a speedy examination of the parts is all important in disputed cases.

A comparison of the sexual organs may be necessary, as the accused may allege, in his defence, that he is impotent, either from disease, a surgical operation, or from defective organization. The body of the male should also be carefully inspected, as it can scarcely be presumed, where the resistance has been considerable, that he should have escaped without scratches or bruises.

Doubts have been intimated by various writers, whether a rape can be consummated on a grown up female, in good health and strength. The opinion of medical men generally is very decisive against it, and Dr. Beck admits the possibility of its occurrence only under peculiar circumstances—as where the female, laboring under great fear or terror, or under the dread of instant murder, is incapable of making much bodily resistance. We perfectly agree in this opinion, and we state it as our firm belief that, where a female is violated by a single man, and in the perfect possession of her corporeal and mental powers, she is more to be blamed than her ravisher.

A diseased state of the vulva, or orifice of the vagina, may occasionally induce appearances not unlike those resulting from rape or external violence. Of this the memorable case of Jane Hampton, reported by the celebrated Dr. Percival, affords a striking example. She was four years of age, when she was admitted as an out patient of the Manchester Infirmary, in February, 1791.

"The female organs were highly inflamed, sore and painful; and it was stated by the mother, that the child had been as well as usual, till the preceding day, when she complained of pain in making water. This induced the mother to examine the parts affected, when she was surprised to find the appearances above described. The child had slept two or three nights in the same bed with a boy fourteen years old, and had complained of being very much hurt by him durng the night. Leeches and other external applications, together with appropriate internal remedies, were prescribed; but the debility increased, and on the 20th of February the child died. The coroner's inquest was taken, previous to which, the body was inspected, and the abdominal and thoracic viscera found free of disease. From these circumstances,

[illegible] this case, was induced to give it as [illegible]th was caused by external violence; [illegible] accordingly returned against the boy [illegible] Not many weeks elapsed, however, before [illegible]ccurred, in which there was no reason to sus[illegible]al violence had been offered, and some in which it wa[illegible]solutely certain that no such injury could have taken place. A few of these patients died. Mr. Ward was now convinced that he was under a mistake in attributing the death of Jane Hampson to external violence, and informed the coroner of the reasons which induced this change of opinion."—*p.* 119.

An inspection of the linen may sometimes be necessary, and hence the importance of being able to distinguish the various stains which may be present. Until recently, this subject almost escaped the attention of the medical jurist, and we are much pleased, therefore, that Dr. Beck has noticed it at some length. A few years ago, we published in the "Western Medical Gazette," a translation of the article on this subject in Orfila's elaborate work on legal medicine, the same from which the author has obtained the outline before us, and which we beg leave to extract for the benefit of the reader.

"Semen forms, when dry on linen, irregular spots of a light yellow or grayish color; but so indistinct, that frequently it is necessary to hold them between the eye and the light to discover their presence. On pressing them with the fingers, the linen appears as if starched. When dry, they are inodorous; but as soon as they are moistened, the spermatic odor is given out. If the linen be gently heated, they assume a yellow fawn color, and this, indeed, will indicate spots which otherwise would pass unnoticed. This property is important in distinguishing the discharge. And it is also found, if the linen be left for some time in distilled water, the above result will not be reproduced on heating it. The semen has become mixed with the water—and no change of color is occasioned.

"In water, the spots become completely moistened; which is not the case, if they have been caused by grease: and on being rubbed, give out their peculiar odor. The fluid itself is of a flocculent, milky appearance, and on being evaporated, is found alkaline, and assumes a mucilaginous appearance; and if the process be continued to dryness, it leaves a semi-transparent residue, resembling gum arabic, and of a light fawn color. This again is decomposable in distilled water, if the mixture be shaken, into two parts; one soluble, but the other glutinous, insoluble in water, but soluble in potash. The soluble portion yields a white flocculent precipitate with alcohol, chlorine, acetate of lead, or corrosive sublimate. Pure nitric acid gives it a slight yellowish tint, *but does not render it turbid.*

Alcohol dissolves but a very trifl[illegible] above, be left in it for twenty-four hou[illegible]

When blenorrhagic matter, obtained [illegible] treated in a similar manner, the linen too[illegible], but the spots do not become yellow, when held [illegible] culiar odor is wanting, but the solution is also alkal[illegible] orated, the product is of a white yellowish color, opake and [illegible] posable by heat. It dissolves with difficulty in distilled water, but alcohol and the other re-agents already named, yield a white precipitate; and *nitric acid also a white one.* Leucorrheal matter wants many of the characters of the spermatic fluid, and the re-agents cause but a slight precipitate, if it be treated in the same manner as already described."*—*p.* 127, 128.

### PREGNANCY.

The chapter on Pregnancy occupies fifty-three pages, and is treated, of course, with the author's accustomed ability and minuteness. The subject is one of no little importance in legal medicine, as upon its proper decision may depend the property, the honor, or even the life of the female. This condition may either be *pretended* or *concealed*, or it may be *imputed* from a variety of motives. A woman, for example, may have a strong desire to gratify the wishes of her husband, and for this purpose she may allege that she is with child; or she may pretend that she is in this state for the purpose of retaining property, to delay the execution of punishment, to excite compassion, to extort money, or to induce promise of marriage. Of the latter, a curious instance is to be found in the admirable treatise of Capuron. A young girl, engaged to be married, simulated pregnancy, and pretended to have been delivered, in order to obtain from her lover the execution of his contract. The trap thus set, however, came nigh proving fatal to the wily designer; for the child, on being claimed, could not, of course, be produced, and it was only, after much exertion that the unfortunate female was cleared of the charge of infanticide, which had been preferred against her.

Again, pregnancy may be concealed from a wish on the part of the female, to destroy her offspring, to place it in the street, or to send it to some hospital; or, finally, it may be imputed

* Orfila, Lecons, 2d edition, vol. 2, p. 573, translated by Dr. Gross, in Western Medical Gazette, vol. 2, p. 244. Sedillot, p. 93.

from a desire to injure her reputation, or to prevent her from getting married.

Such are a few of the cases which may require judicial investigation, and in which a knowledge of the signs of pregnancy is so essentially necessary. "For want of clear notions on the subject of distinguishing utero-gestation from other affections, and for want of a very attainable degree of tact, practitioners," says an eminent writer,* "are frequently incurring disgrace, and patients are subjected to active courses of medicine for the reduction of tumors, for which the natural remedy is parturition."

The signs of utero-gestation may be distinguished into two classes, the *rational* and the *sensible*; the former resulting from the influence which the uterus exerts upon the physical and moral system of the female, the latter from the enlargement of the womb and the presence of the fœtus.

The rational signs of pregnancy consist, first, in the suppression of the menses; secondly, enlargement of the abdomen, thirdly, increased size of the breasts, attended with a lactiform or watery secretion, and a change of color in the skin of the nipple; fourthly, loss of appetite, nausea, vomiting, and frequent spitting; fifthly, the state of the pulse and the properties of the blood; and sixthly, a change in the moral and intellectual faculties.

We shall not stop to assign to each of these phenomena the share of confidence which it may deserve in deciding on the presence or absence of pregnancy; suffice it to say, that they are all fallacious, and therefore, taken apart from the sensible signs, perfectly valueless.

Many ludicrous mistakes have occured in relation to the diagnosis of abdominal tumors. The case mentioned by Sir Astley Cooper, of a young physician who tapped a lady for "dry dropsy," is too familiar to the reader to be repeated in this place. Dr. C. of Philadelphia, is said to have committed a similar blunder; and we have heard of not less than two or three, of a well authenticated nature, which occurred in the

* Dr. Gooch.

eastern part of Pennsylvania, and in which tapping was near being performed, although the proper cure was parturition. In one of the cases referred to, the woman was supposed to be laboring under abdominal dropsy, for the removal of which she was put on the use of the ordinary hydrogogue medicines. Finding no relief, however, from the prescriptions, and the enlargement of the abdomen daily augmenting, the medical attendant stated to the family of the patient the necessity of performing the operation of paracentesis, and for this purpose he desired the counsel of a professional brother. The day was fixed for the operation; the physicians arrived, but on entering the rcom, and inquiring after the health of their patient, their dismay can be better guessed than described when the nurse presented them with a fine healthy child, by saying, "doctors here is the dropsy!"

The *sensible signs* of utero-gestation, those on which alone reliance can be placed, consist in the changes experienced by the mouth, neck, and body of the uterus, in the active and passive movements of the fœtus, and in the detection of the fœtal and placental sounds by means of the stethoscope.

The alteration of the state of the uterus, ascertained by what is technically called the *touch*, is thus explained by the author:

"After conception, the fundus and body of the uterus both increase, and the former, from its becoming heavier, will naturally descend lower down in the pelvis, and thus project farther into the vagina. The uterus remains in this situation until it becomes so large as to rise out of the pelvis; and accordingly this temporary abbreviation of the vagina, is a sign of pregnancy, though, of course, an equivocal one. The body of the uterus enlarges. The changes in the neck are also striking. In the unimpregnated state it projects into the vagina about two thirds of an inch, like a thick, firm and fleshy nipple. At the termination of pregnancy, this neck is completely obliterated; the portion of uterus which lies over the top of the vagina, no longer projecting into its cavity, but forming a flat roof.

"This obliteration generally commences in a first pregnancy, about the fifth month, but in females who have had several children, the neck yields more readily, and accordingly with some, it as much altered at the fourth, as it is in the previous case at the sixth month.

"During the period of these alterations, the vagina is more elongated, since the uterus rises farther up. But towards delivery, this viscus gradually re-descends. The os uteri also varies with the changes in the cervix. The lips gradually flatten and disappear, and towards delivery, a small rugous hole only is discoverable."—*p.* 174.

The most infallible sign, undoubtedly, of utero-gestation, is the detection of the fœtal and placental sounds by means of auscultation. This subject was first distinctly noticed by Dr. Kergaradec, of Paris, in a paper read before the Royal Academy of Medicine in 1821, and published in 1822. It has since attracted the attention of numerous physicians, both in Europe and in this country, and may be justly regarded as one of the most brilliant and important discoveries of the present day. The following remarks are too valuable and instructive to be passed unnoticed.

"The indications of the presence of a living fœtus in the womb, as derived from auscultation, are two: 1. *The action of the fœtal heart.* This is marked by double pulsations, and it greatly exceeds in frequency the maternal pulse. In the first case noticed by Kergaradec, it varied from 143 to 148 in a minute, while the pulse of the mother was not more than 70. These pulsations may be perceived as early as the fifth, or between that and the sixth month. Their situation *varies with the position of the child*, and accordingly they are more distinct at one time than another, in the same place, and in different places at different times. Their most general situation, however, is the lower part of the abdomen. The space over which they are perceptible at the later period of pregnancy is about a foot long, and three or four inches broad, and their intensity of course, corresponds with the nearness of the observer to the source of the sounds. In the early months they are necessarily less manifest in each respect. The fœtal circulation does not appear to be affected, in health, by agitation in the maternal. It varies from 120 to 160 in a minute, always far exceeding, as already stated, that of the mother. The only opposite case ever noticed, was that by Dr. Ferguson, in which the fœtal heart was distinctly heard to beat only 28, and the mother's 100. From its rareness it is possible that some peculiarity in structure may have been the cause. Dr. Kennedy, however, relates instances in which the mothers were laboring under disease and the loss of blood, either by hæmorrhage or venesection, produced striking changes in the fœtal circulation.

"2. The second auscultatory sign of the presence of the fœtus has been variously termed the *placental sound*, the *placental bellows sound*, and the *utero placental soufflet.* It is generally agreed that its seat is in the enlarged vessels of that portion of the uterus which is immediately connected with the placenta. Lænnec remarks, that it is evidently an arterial pulsation perfectly isochronous with the pulse of the mother, and accompanied by a rushing noise resembling the blast of a pair of bellows. The place which it occupies never changes, but it varies in different individuals, and is seldom so large in extent as the space in which the fœtal heart is perceptible. The time at which it commonly begins to be heard, is the fourth month, or as soon as the fundus of the uterus has risen above the upper brim of the pelvis, so that it can be brought in contact with the abdominal parieties, by the pressure of the extremity of the stethoscope. It is said to be even louder then, than at the full term. Certainly at later periods the sound is duller, more diffused, and no longer gives the sensation of being confined to a single artery. Dr. Ferguson observes, that he has most frequently found the placental sound in either iliac region, although he has detected it in almost every part of the abdomen.

"The noise of the placenta, and the action of the fœtal heart, are commonly found on opposite sides of the body. This, however, is not constantly the case, for sometimes both the phenomena are audible on the same side; and in one case Lænnec and Kergaradec perceived the heart's action behind that of the placenta—the place where they were examined, being the interior part of the hypogastric region." —*p.* 176.

### DELIVERY.

The medico-legal questions in relation to delivery, may be reduced to three. First, Are there any signs by which it can be ascertained that a woman has been recently brought to bed, and, if so, how long after the event is it possible to discover traces of it? Secondly, Can a woman be delivered unconsciously? and Thirdly, When the mother and infant are both found dead, which of them may be considered the survivor? The decision of these questions necessarily involves an examination of the signs of delivery, together with several other circumstances that will be referred to in the course of these remarks.

"If the female be examined within three or four days after the occurrence of delivery, the following circumstances will generally be observed: Greater or less weakness, a slight paleness of the face, the eye a little sunken, and surrounded by a purplish or dark-brown colored ring, and a whiteness of the skin, like a person convalescing from disease. The belly is soft, the skin of the abdomen is lax, lies in folds, and is traversed in various directions by shining reddish and whitish lines, which especially extend from the groins and pubis, to the navel. These lines have sometimes been termed *linæ albicantes*, and are particularly observed near the umbilical region, where the abdomen has experienced the greatest distension. The breasts become tumid and hard, and on pressure emit a fluid, which at first is serous, and afterwards gradually becomes whiter; and the presence of this secretion is generally accompanied with a full pulse and soft skin, covered with a moisture of a peculiar and somewhat acid odor. The areolæ round the nipples are dark colored. The external genital organs and vagina are dilated and tumefied throughout the whole of their extent, from the pressure of the fœtus. The uterus may be felt through the abdominal parities, voluminous, firm and globular, and rising nearly as high as the umbilicus. Its orifice is soft and tumid, and dilated so as to admit two or more fingers. The fourchette or anterior margin of the perinæum is sometimes torn, or it is lax, and appears to have suffered considerable distension. A discharge (termed the lochial), commences from the uterus, which is distinguished from the menses by its pale color, its peculiar and well known smell, and its duration. The lochia are at first of a red color, and gradually become lighter until they cease.

"These are the signs enumerated by the best writers on the subject, and where they are all present, no doubt can be enteriained that delivery has taken place.—*p.* 207.

The signs here enumerated, taken separately, cannot, it is obvious, afford positive proof of recent parturition; considered jointly, however, and in connexion with other circumstantial facts, they afford abundant reason for concluding that there has been delivery. Professor Chaussier, of Paris, states that there is no disease or affection besides parturition, which can produce the whole series of phenomena described by the author, and in this opinion we feel disposed perfectly to coincide.

All examinations of this kind should be instituted as early as possible after the event has taken place, otherwise great difficulty must necessarily be experienced in coming to a satisfactory decision. As a general rule, it may be stated, that after the fifth or sixth day, the signs are already considerably less evident; and at the end of the seventh or eighth, but few can be observed. "After the tenth day, the signs become equivocal, and may lead to error, particularly if the delivery has been natural."

Instances occasionally occur where the woman dies shortly after a supposed or pretended delivery; and here, of course, the body should always be examined, in order to ascertain the truth. If the inspection be made soon after the event has taken place, the uterus will appear like a large flattened pouch, from six to eight or ten inches in length, and containing more or less blood, either in a fluid state, or, as is more generally the case, in the form of coagula. The marks of the attachment of the placenta will be very visible, and the uterine appendages, especially the Fallopian tubes and ovaries, will be of a dark purplish color, owing to the fact that these parts are always very vascular during utero-gestation. A week after delivery, the womb is as large as two fists, and at the end of a fortnight, it will be found almost six inches long. At this period, the organ usually lies obliquely to one side, its inner surface is still rough and bloody, and the orbicular direction of the fibres round the orifice of the Fallopian tubes, is very evident. These appearances frequently continue for three or four weeks before the uterus returns to its natural state. In addition to these phenomena, the vagina is usually relaxed, the broad ligaments of the womb are diminished in size, the skin of the abdomen lies in loose folds or wrinkles, and there will be one or more *corpora lutea*, or yellow bodies.

The corpora lutea are oblong glandular bodies, of a dusky buff color, formed by a rupture of the ovarian vesicles. In the early stages of pregnancy, as well as for some time after delivery, they are extremely vascular, excepting at their centre, which is whitish, and which contains a small cavity, supposed to be produced by the escape of the ovum. In many instances they are of an oval stellated form, looking a good deal like a radiated cicatrix. These bodies gradually fade and wither; but there is no regularity as to the time of their disappearance.

It was formerly a subject of much controversy amongst physiologists, and indeed the question can scarcely be said to be perfectly decided even at the present moment, whether corpora lutea ever exist independently of impregnation. The great Haller, who was the first to point out these little bodies, positively asserts, that their presence affords incontestible proof of previous conception; and the same opinion has since been warmly espoused by other writers, especially the British. In the celebrated case of Charles Angus, esquire, who was tried in 1808, at the Lancaster assizes, for the murder of Miss Burns, the most eminent physiologists of London, as Denman, Haighton, Clarke, Abernethy, and [Sir] Astley Cooper, maintained, almost without exception, that the corpus luteum, which was found in one of the ovaries of the deceased, could alone be explained on the supposition of an advanced pregnancy. The venerable professor Blumenbach, of Gœttingen, seems to have been the first who suggested the idea that these bodies may occasionally exist without impregnation, simply as the result of strong sexual excitement. The hint thus thrown out, induced Sir Everard Home shortly afterwards more fully to investigate the subject, and his researches make it very clearly appear, that the corpora lutea cannot be regarded, under all circumstances, as a positive sign of pregnancy. His observations were made on several young girls, who died during the virgin state, and in whom the hymen was too perfect to admit of the possibility of impregnation. In all these cases, he observes, there were not only distinct corpora lutea, but also small depressions round the edges of the ovary, caused evidently by the rupture at a former period of some of the vesicles of De Graaf. The results of these researches have been confirmed, more recently, by the experimental inquiries of Dr. Haighton and Dr. Blundall; and, taken in connexion, they may be considered, we think, as having finally set the question in great measure at rest. These distinguished physiologists performed their experiments on rabbits, which they allowed to be near each other during heat, without permitting them, however, to copulate. After the sexual excitement had passed away, the doe was killed, and although she never had any connexion with the male, her ovaries presented several well-marked corpora lutea. These experiments were frequently repeated, and generally with similar results.

Very recently the inferences deduced from these observations, have been called in doubt by professor Montgomery, of Dublin. From a large number of personal examinations both in the human subject, and in many of the inferior animals, as the cow, mare, sheep, sow, bitch, goat, hare, cat and rabbit, he expresses it as his firm conviction that the real corpora lutea *never occur without previous impregnation.* He supposes that those who have asserted that these bodies may exist without conception, and of course to be found in the virgin ovary, have been led into the error by confounding appearances and structures essentially different, and in fact having only one character in common, which is their color, altogether forgetting that "every yellow substance in the ovary is not a corpus luteum."

The virgin corpus luteum differs, according to Dr. Montgomery, in many respects from the real one. In the first place there is no external prominence or cicatrix; secondly, it is not vascular, and consequently cannot be injected; thirdly, it does not present that soft, glandular aspect so characteristic of the real yellow body; fourthly, there is no central cavity, nor the star-like cicatrix which results from its closure; and fifthly, one or more of these bodies often occur in both ovaries at the same time, especially in females who die of tubeucular disease of the lungs and other organs.* These observations are exceedingly interesting, and emanating as they do, from a man of large experience, and great acuteness of intellect, they must be received as worthy of peculiar confidence. Should they be true—and this, we conceive, can only be determined by future researches—they will present the question which we have been examining in a very different light from what it has been viewed by some of the British physiologists. The subject is one of vital importance in juridical medicine, and we sincerely trust that the readers of this journal will use their best efforts to contribute something towards its elucidation.

Can a woman be delivered unconsciously? Certainly she can, especially if she be intoxicated, comatose, delirious, idiotic, or laboring under an attack of apoplexy, or violent hysterical convulsions. About five years ago we delivered a young woman of twins, who, when we announced to her after the first child was born, that there was another, immediately fell into a state of hysteria; on recovering from which she was perfectly unconscious of what had happened. A more remarkable instance of this kind occurred some years since in Easton, Pennsylvania. Mrs. I., the wife of a member of Congress, was taken in labor, but before the child was born she was attacked with puerperal convulsions, from the ef-

* London Cyclopædia of Practical Med. Art. Pregnancy. p. 502.

fects of which she suffered for upwards of four weeks. At the expiration of this period, she one day suddenly recovered her reason, and finding her abdomen perfectly flat, anxiously inquired what had become of her infant, having all this time remained perfectly ignorant of her real situation. Several cases of a similar nature are referred to by our author, and others are to be found in the writings of Cheyne, Capuron and Montgomery. These examples prove that it is possible for a female to be delivered without her knowledge; but the circumstance is so extremely rare that, as a general rule, the assertion should be disbelieved.

The third and last question upon which we have proposed to comment is, "when both the mother and child perish, who was the survivor?" Concerning this question—which is one of much interest in relation to the inheritance of property—various opinions have been promulgated by writers on forensic medicine, some supposing that the mother, others that the infant always dies first. The former of these views was entertained by the imperial chamber of Wetzlar, who decided in one or two important cases that the death of the woman uniformly precedes that of the infant. The reasons for this decision were grounded on the belief, first, that parturition, when unassisted, generally exhausts the strength of the female; and secondly, on the assumption, apparently well founded, that the child only perishes, when, in consequence of the death of the mother, it is deprived of its usual aliment. On the other hand, we have the authority of Valentin, Capuron, and other highly respectable writers, in favor of the opinion that the child, and not the mother, is the one that usually dies first; their arguments resting on the belief that the tender and delicate organs of the fœtus are always more or less compressed during difficult parturition, and hence, that, however painful and protracted may be the labor, the strength of the mother is never exhausted before that of the child. The latter of these views, is, perhaps, the more philosophical of the two; for it must be admitted, we think, for a vast variety of reasons which will readily suggest themselves to the reader, that, in the great majority of cases, the infant will be likely to perish before the unhappy being who is unable to give it birth.

We had intended, in connexion with this subject, to make some remarks on the development of the fœtus, and the viability of the infant, but time admonishes us to "speed our pen," and we therefore content ourselves by referring to the work itself where both these topics are treated at great length, and with much ability.

### INFANTICIDE.

We now come to one of the most important chapters in the whole book, involving the consideration of a subject which, both in this country and in Europe, becomes the topic of medico-legal investigation more frequently, perhaps, than any other in the whole range of the science. Hitherto, the reader will please to bear it in mind, we have been speaking exclusively of the labors of Dr. Theodric Romeyn Beck, and as he is the author of the greatest part, by far, of the work before us, we have invariably used the singular number, although two names appear on the title-page. The portion of the "Elements" which is now about to engage our attention, is from the pen of Dr. John B. Beck, to whom all the extracts and remarks that may be made under the head of infanticide are alone to be referred. The propriety of bearing in mind this fact, will appear in its true light, when it is remembered that no man is entitled either to the applause or to the censure of another.

The crime of infanticide, revolting as it is to the best feelings of our nature, has probably prevailed, to a greater or less extent, in every nation, civilized as well as barbarian, from the earliest ages of the world until the present moment. Amongst the Canaanites, nothing was more common than to sacrifice children to the idols of their country; and amongst the ancient Persians, it was a common custom to bury children alive. In most of the Grecian states, infanticide was not merely permitted, but actually enforced by law; an injunction defended by some of the ablest men of the country. A similar practice was in vogue amongst the ancient Romans, who treated their infants with the most unrelenting cruelty. Romulus authorized the destruction of all infants that were deformed; and in subsequent times it became the custom of many parents to expose their children to wild beasts and to birds of prey, or to strangle them in the most horrid manner. Provoked abortion seems also to have been very common, and is thus mentioned by Juvenal:

> "Such is the power of herbs; such arts they use
> To make them barren, or their fruit to lose."

But child-murder was not confined to the ancients. It has descended to modern nations, and, until very recently, was practised to an enormous extent in China, and in most of the south sea islands. We are informed by Mr. Ellis, in his Polynesian Researches, (vol. 1, 1833), that societies formerly existed in these islands, which not only sanctioned the destruction of infants, but imposed on their members the express injunction that no child should be permitted to live. It is asserted that, on the arrival of the first European missionaries at the Sandwich Islands, the disproportion between the infants spared, and those destroyed, was truly appalling, not less than two-thirds of them, it is supposed, having been murdered by their own parents. Fortunately, this crime is at present much less common than it used to be, and there is much reason to believe that the spread of Christianity, whose mild and humane spirit is so completely averse to it, will ere long oppose an effectual barrier to its further progress throughout every heathen land.

Our analysis of this chapter—which embraces the enormous extent of one hundred and seventy-seven pages—must necessarily be confined to the more important topics of which it treats, and amongst these we propose to dwell with some degree of minuteness upon the various tests that have been suggested for the purpose of detecting the crime in question, together with a brief examination of some of the more prominent objections that have been urged against their validity.

The proofs of the survival of the child after delivery, must be drawn mainly from the changes which take place in the respiratory and circulating organs, in consequence of the blood being directed into other channels. These changes relate principally to the color, size, consistence, and specific gravity of the lungs; to the state of the ductus venosus; to the closure of the foramen ovale; and to the contraction of the ductus arteriosus, situated between the pulmonary artery and the arch of the aorta.

The very first thing to be done by the physician, when called upon to make an examination of the body of a dead infant, is to note the precise spot where it is found; the kind of clothes or dirt by which it is covered; the state of the umbilical cord; and such bruises, ecchymoses or lesions as may exist upon its exterior. The body should also be weighed, and especial care taken to ascertain whether the umbilicus be at or near the middle of the entire length, as this, in connexion with the state of the nails and hair, will materially assist us in determining its age. After attending to all, even the most trivial appearances, to be observed externally, the examiner should proceed to inspect the internal organs.

In the fœtal state, the thorax is flat and compressed, the diaphragm is arched, and the lungs are comparatively small in volume. As soon, however, as respiration takes place, these organs become distended with air, and, as might be expected, the cavity of the chest is greatly enlarged in all directions. During intra-uterine life, the lungs are situated in the recesses formed by the ribs and spinal column, the left one being partially concealed by the thymus gland; they do not cover more than about one-half of the superior surface of the diaphragm, nor do they embrace, as is the case after breathing has been established, the sides of the pericardium. The color of the fœtal lungs is stated by Dr. Beck, to be *brownish red.* With this our own experience does not accord, having found these organs, in most cases, of a *light purplish tint.* After respiration has taken place, they become of a scarlet or pale red, at least those parts of them that have been penetrated by air.

The posterior surface of the lungs, it should be observed, is generally of a darker color than the anterior, especially if the child have lain for some time on its back, owing to the subsidence of the blood. It would seem from the experiments of professor Bernt of Vienna, that artificial inflation of the lungs of a dead child usually causes a greyish red tint, very different from the scarlet tint of natural respiration; though occasionally no change whatever takes place.

In regard to their consistence, the fœtal lungs are always more or less compact, being not unlike the dense and solid substance of the liver; as soon, however, as breathing takes place, they become soft, spongy, crepitate on pressure, and emit air bubbles or froth on being squeezed with the hand.

In the fœtus, anterior to respiration, the lungs are always specifically heavier than water, and hence they uniformly sink in that fluid; after respiration, however, has been established, they become specifically lighter, and therefore float on its surface. It is upon a knowledge of these facts—first noticed by Galen, but not applied to the purposes of forensic medicine until a short time after the middle of the 17th century—that is founded what is termed the *hydrostatic test*, or, as it is more commonly called on the continent of Europe, the *pulmonary docimacy*. "There is not," as has been justly observed by Dr. Beck, "in the whole range of forensic medicine, a question more important, and at the same time more difficult, than the one which relates to the floating of the lungs as a proof of the child's having been born alive. It has divided the opinion of medical jurists from the earliest periods, and even at the present day, it still remains the subject of controversy." The different objections that have been urged against the conclusiveness of the hydrostatic test, have been arranged by the author of the chapter on infanticide into two classes; the first, embracing those which go to show that the lungs may float, and yet the child not have been born alive; the second, those which go to show that these organs may sink in water, and yet the child may have been born alive.

The first objection examined by Dr. Beck is the floating of the lungs in water, from the evolution of air in consequence of putrefaction. This circumstance was first noticed more than one hundred years ago by the celebrated Heister, who observed the lungs of a child which had died in the womb, to float in water, both entire and when cut in pieces. Haller, Camper, Jaeger and Mayer, afterwards instituted experiments on the subject, and more recently it has received important contributions from the labors of Orfila, Devergie, Beck, and other writers. The most satisfactory observations, perhaps, are those of Mayer.

"From a very extended series of experiments, continued during a number of years, and executed with the utmost care and precision, Mayer found, on putting into water the lungs of still-born children, in whom, of course no sign of respiration or life had appeared, that they sunk to the bottom. After an interval of two or three days, the water in which they were left became turbid—the lungs changed in color, and increased in volume—here and there an air bubble arose to the surface of the water, and at the same time a putrid odor became perceptible. All these appearances continued to increase daily, until the sixth, seventh, or, at the latest, the eighth day, when the lungs, both entire and cut into pieces, floated in the water in which they became putrid. On transferring the lungs to vessels containing clean water, they still continued to float, although on the slightest compression they instantly sunk.

"Lungs placed in water, and exposed to the rays of the sun, swam on the sixth day. If they were suffered to putrefy where there was a free current of air, they rarely floated before the tenth or eleventh day. After the lungs had once floated, they remained in that state, emitting daily a more offensive odor, and acquiring an increased volume, until the twenty-first, or at the latest, the thirty-fifth day. After that period, they gradually sunk down, without a single exception, to the bottom of the vessel, nor did they afterwards betray any disposition to float, although kept for seven weeks, and in some instances a much greater length of time.

"The foregoing experiments were made in the month of August. The lungs, both entire and cut into sections, were immersed in pure fountain water, and contained in vessels convenient and capacious. In short, every precaution seems to have been scrupulously observed, to render the experiments accurate and satisfactory.

"My own experiments on this subject, although not numerous, go to confirm in every essential point, those which have been just detailed."—*p*. 366, 367.

It may not, perhaps, be uninteresting here to add the results of some experiments which we performed on this subject in

1832, more especially as they are somewhat at variance with those of Mayer, relative to the period at which the lungs began to float in water. In the month of July, the right lobe of a still-born infant was placed in an open glass vessel, and exposed to the rays of an intensely hot sun. At the end of twenty-four hours, it was found to swim on the surface of the water; there was a large air vesicle under the pulmonary pleura; and the whole organ was expanded and offensive. In twelve hours more the lung was extremely fœtid; numerous air-bubbles were scattered over different parts of its surface; and in seventy-two hours after the experiment was begun, it still floated, both in the water in which it was originally immersed, and in the clear fluid.

The left lung, taken from the same child, was kept for twenty-four hours in a dry glass vessel, and then placed, already somewhat offensive, in pure rain water. In twenty-four hours afterwards, the organ was seen floating, presenting here and there an air-bubble. It was then removed from the vessel, and, after being firmly pressed with the hand, it was replaced in clean water, when it immediately sunk to the bottom of the fluid. In six hours after this, however, it again rose to the top, and on renewing the compression eighteen hours subquently, it was quite impossible, with all the manual pressure that we could command, to squeeze out the air so as to make the organ sink.

In these experiments, which have been several times repeated, the floating of the lungs, it will be observed, took place much sooner than in those of Mayer. Whether this physician exposed the lungs to the rays of the sun, or kept them immersed in water, standing in the shade, it is not easy to determine, as no mention is made of the circumstance, yet this was no doubt the case, inasmuch as the heat of July or August is probably nearly or quite the same in Germany and Ohio.

Dr. Beck supposes that the changes produced in the lungs by putrefaction, may always be readily distinguished, amongst other marks, by the peculiar appearance of the vesicles on the surface of these organs, and by the ease with which the air can be extricated from their texture, so as to make them sink in water.

Jaeger long ago observed that the evolution of gas always begins underneath the pulmonary pleura, where the air introduced by respiration never finds its way. Similar observations have since been made by Dr. William Hunter, who describes the air bubbles as running in lines along the fissures between the component lobules of the lungs. These little vesicles, according to our own observations, vary in size from the smallest pin's head to that of a little pea; they have a whitish or greyish appearance, looking like so many little pearls, and they may be readily pushed about from one place to another by the pressure of the finger.

The opinion, that the air, caused by putrefaction, can be easily extricated from the lungs, so as to make them sink in water, seems to be very generally entertained by medico-legal writers. Dr. Beck appears to give it full credence, as the following extract will show. "The evidence of this is to be found in the fact, that if lungs of this description, or any portions of them, be squeezed in the hand, they will immediately sink in water. On the contrary, no compression, however strong, can force out so completely the air from the lungs that have respired, as to cause them to sink. This test is insisted upon by Marc, a very distinguished writer on this subject, as the most certain means of discriminating between the effects of putrefaction and respiration."

Now we are by no means so certain that the air can be so easily squeezed out as is imagined by Dr. Beck, and his French authority, Dr. Marc. In the experiment before alluded to, in which the left lung was kept for 24 hours in a dry vessel, and afterwards immersed for two days in water, we were perfectly unable, with all the force we could command, to rid the organ of air, so as to compel it to sink; and we are inclined to believe, from this and other trials, of a similar nature, that, when the evolution of gas is complete, or, in other words, when it pervades the entire substance of the lungs, it will be *utterly impossible* to prevent them from floating.

Where any doubt, however, remains concerning the real cause of the floating of the lungs, the difficulty may, in general, be easily removed by an examination of the other viscera; for numerous observations have established the fact that, with the exception of the bones, the lungs resist putrefaction longer than any other part of the body.

It has been objected, secondly, that a child may have been born dead, and yet its lungs, in consequence of artificial inflation, swim in water; whence it has been alledged, that the mere floating of these organs is no proof of natural respiration.

"It has been doubted by some, whether artificial inflation of the lungs can ever be effected. Heister states that he proved, by actual experiments, that air cannot be blown into the lungs so as to cause them to float. Hebenstreit also doubts whether it can be accomplished, in consequence of the mucus which is usually found to fill the fauces of a new-born child. Roederer, from the failure of his experiments on this subject, was led to the conclusion, that it can only be effected after the child has previously breathed. 'A spirtu ori,' says he, 'inflato pulmones infantis non inflari dilatarique; nisi fœtus aliunde respiraverit.' Brendel is still more positive on this point. He believes artificial inflation to be utterly impossible, and assigns two reasons for his scepticism. The first is the resistance which is made by the thorax and diaphragm; and the second is the difficulty of introducing a pipe into the glottis, without which he thinks it is impossible to inflate the lungs. He adds, moreover, in confirmation of his opinion, that he made experiments upon pups that were killed while yet in the uterus; and although he attempted to force in the air by a bellows, yet no change was effected upon the lungs, and they sunk when put into water.

"A contrary doctrine is, however, maintained by a very large majority of the most respectable authorities in forensic medicine. Low admits the possibility of it, and tells us that Bohn, together with the medical faculty of Leipsic, concurred in the same opinion. Ludwig says, it is certain that air may be artificially blown into lungs which have never respired, and that they will afterwards float in water. In several experiments made to test this matter by the celebrated Camper, the result was uniformly in favor of this opinion. Jaeger, Buttner, and Schmitt, concur in the same, as do most of the French and English writers. Dr. Gooch says he inflated the lungs of a still-born child, and they floated in water as if the child had breathed some days.

"From the foregoing detail of authorities, it is quite evident, that although artificial inflation of the lungs of a child born dead, is a thing perfectly practicable, yet it is not accomplished with as much facility as many have imagined."—*p.* 370, 371.

We are decidedly of opinion that artificial inflation of the lungs is a very difficult matter; and we believe that the complete distention of these organs can only be effected where a tube is introduced into the mouth of the larynx. A case which recently came under our notice, greatly corroborates this opinion. Here the child was still-born, in consequence of the delay occasioned by a mal-presentation; and although repeated efforts were made by our friend Dr. Ezra Read, the attending physician, to resuscitate the infant, yet we found on examination that only a small portion of the right middle lobe, together with a few lobules of the right superior and right inferior lobes, was filled with air.

It has been objected, thirdly, that the lungs may float in water, simply by being emphysematous, in consequence of violence done to them during delivery. In these cases, which must of course, be very rare, the aeriform fluid exists only in the interlobular cellular tissue, from which, according to Chaussier, it may be readily squeezed by the pressure of the hands.

The fourth objection examined by Dr. Beck, and one that may be regarded as of no little interest in connexion with the hydrostatic test, is, whether the child is capable of breathing as soon as its mouth is born, or protruded from its mother, and if so, whether it may loose its life before the expulsion of the rest of the body? On this subject, we prefer to quote the author's own language.

"This objection did not originate with Dr. Hunter. It is noticed by Morgagni, and I find it discussed by the German writers early in the last century. It must be admitted, however, that the high authority of Hunter's name has given to it an importance which it otherwise would never have possessed, and it is on this account more especially deserving of examination. It involves two points, each of which is worthy of distinct elucidation. Is it possible that a child can breathe, when nothing more than its head is delivered? and if so, is it probable, that after having respired in this situation, it will die before the delivery of the rest of the body."—*p.* 374.

Having cited these authorities, Dr. Beck goes on to say; "it must, therefore, be conceded that a child may breathe and cry as soon as its head is delivered, although it is equally true, that it is by no means a common occurrence." Those who may wish for further information on this subject, will find it fully discussed in an article on "Intra-uterine Respiration, in its relations to Physiology and Medical Jurisprudence," in the July number of the Western Medical Gazette, for 1834, where not less than four cases are adduced as having occurred in our own practice, in which the child breathed as soon as its head was protruded from the vulva of themother; and since the publication of that paper, the same thing has been observed by us in nearly as many more. We have reason, therefore, to believe that this occurrence is much moie common than has been imagined by Dr. Beck and other jurists.

A fifth objection, noticed by the author, is founded on the fact that a child may occasionally breathe while yet in the womb, before any portion of it is delivered. That this occurrence may happen after the rupture of the fœtal envelopes, there cannot be the slightest doubt, notwithstanding the assertions of some able physiologists to the contrary; yet that it is very rare, is equally true. Bredenoll and Bernt, two highly respectable German writers, relate cases of it as having come under their own observation; and more recently, professor Holmes, of Montreal, in Canada, Dr. Kennedy, of Dublin, Mr. Tompkins, of England, and the editor of the London Medical Review, have published instances of this species of respiration. In answer to this objection, Dr. Beck very properly observes that such cases must be extremely rare, and that, even granting that they may take place, the inflation would necessarily be so imperfect that the lungs would either partially or completely sink in water.

Besides these causes, the lungs after having been inflated with air, may sink in consequence of disease, as tubercles, or inflammation, or from a want of ability on the part of the infant to draw in a sufficient quantity of air to distend these organs to such a degree as to compel them to float in water. The former of these causes, however, seldom exist, or even supposing that they did, nothing certainly would be more easy than to detect the presence of morbid structure; and as to the latter—namely, imperfect distention of the lungs, it would be equally easy to ascertain the extent of the aeriform permeation; so that in neither case any real difficulty would be likely to arise.

From a careful, minute, and thorough examination of the several objections that have been urged against the validity of the hydrostatic test, the talented and learned author comes to the following just conclusions:

"1. That when the lungs float in water, it must be from one of four causes: natural respiration—putrefaction—emphysema—the artificial introduction of air.

"2. As the lungs may float from other causes besides respiration, their mere floating is no proof that the child was born alive.

"3. As, however, it is possible to discriminate between the floating of natural respiration, and of that which is the result of other causes, it follows,

"4. That, with due precautions, the floating of the lungs may be depended upon as a decided proof that the child has been born alive."—*p.* 383.

On the other hand, it may be concluded, the author thinks,

"1. That when the lungs sink in water it must be from one or other of the following causes: the total want of respiration—feeble and imperfect respiration—some disease of the lungs, rendering them specifically heavier than the water.

"2. As the lungs may sink from other causes than the absence of respiration, their *mere sinking* is no decisive proof of the child's having been born dead.

"3. As, however, the sinking from the want of respiration may easily be distinguished from that which is the result of other causes, it follows,

"4. That with due precautions, the sinking of the lungs is a safe test that the child was not born alive."—*p.* 388.

The following remarks comprehend an excellent summary of *practical rules*, which should guide the physician when called to the examination of a case of infanticide, which, as is justly observed by the author, demands, of all others, a combination of the soundest judgment and the most profound knowledge.

"(*a.*) As preliminary to any examination of the lungs, the child should be weighed, and the general appearance and condition of the body should be particularly noted, with the view of ascertaining the following points, viz: If the child be full grown, if the different parts of its body be well proportioned; if the shoulders be uncommonly large, when compared with the size of the head; if any tumors are to be found upon the body; if the cord be unusually short; and, finally, if any symptoms of putrefaction be present.

"(*b.*) The chest should then be carefully opened, and the following things noticed: the general shape of the thorax; the situation of the lungs, especially their relative situation to the diaphragm and pericardium—their volume—their shape—their color; and whether there be any appearance of putrefaction.

"(*c.*) The next step is to remove the contents of the chest, for the purpose of performing the necessary experiments upon the lungs. The aorta and vena cava should first be tied near the heart, and then cut beyond the ligatures; the trachea should then be also divided. The lungs, together with the heart, are now to be taken out of the chest, and to be submitted to an additional inspection, to ascertain whether they are sound or diseased, and if they are at all affected by putrefaction.

"(*d.*) A convenient vessel containing water should now be provided, and particular attention should be paid to the temperature of the water in which the lungs are to be immersed. The reason of this will be perfectly obvious, when it is recollected that the specific gravity of water varies with its temperature; thus, for instance, water at 100° is lighter than water at 60°, and still lighter than at 40°. Besides, if the water be too hot, it will have the effect of expanding the lungs, and thus favor their floating, especially when there already exists a tendency to putrefaction. If, on the contrary, its temperature be too low, the air cells may be contracted, and some of the air be thus expelled. The temperature of the water should therefore be regulated by that of the surrounding air. Another precaution relative to the water is, that it should not be impregnated with *salt;* for, in consequence of the greater specific gravity of saline water, a body might float in it, which would sink in fresh water.

"(*e.*) The lungs, together with the heart, should then be cautiously placed in water, and it should be observed whether they float or sink: if they float, whether above the surface of the water, or just under it; if they sink, whether they do so rapidly or gradually.

"(*f.*) The lungs should then be taken out of the water, and after tying the pulmonary vessels, they should be separated from the heart, and accurately weighed.

"(*g.*) The lungs should then be replaced in the water, to see whether they sink or float, and in what way.

"(*h.*) The two lobes should then be separated, and the same experiment repeated upon each, noticing the difference, if any, between them. If one only floats, see if it be the *right* one.

"(*i.*) Each lobe should then be divided into a number of pieces, taking care not to confound the fragments of one lobe with those of the other, and upon each other of these the same experiments should be instituted.

"(*k.*) While cutting the lungs, observe if there be any crepitus; if the vessels are charged with blood; and if there be any traces of disease.

"(*l.*) If any of the sections of the lungs float, they should be taken and squeezed forcibly in the hand, and then replaced in the water, to determine whether after this they will sink.

"Having gone through these different processes, the conclusions to be drawn from them are evident. If there is nothing to be discovered on the body of the child, to favor the belief that it might have lost its life during delivery—if the lungs be not touched by putrefaction, nor be artificially inflated—if on cutting into them, a crepitus be perceptible—if the entire lungs, as well as the separate divisions of them, remain on the surface of the water—if, after squeezing portions of the lungs, they still continue to float—then the mass of evidence is strong, that the infant enjoyed perfect respiration. If only the *right* lung, or its pieces, float, the respiration has been less perfect. If some pieces only float, while the greater number sink, it proves respiration to have been still less complete. On the other hand, if neither the entire lungs, nor any section of them, float in water, the inference is, that the child never respired."—*p.* 388, 389, 390.

The above conclusions, it seems to us, are perfectly held out by the premises; and, however much lawyers andmedical men may talk and cavil about the validity of the hydrostatic test, certain it is that it is the only one upon which the slightest reliance can be placed in the present state of the science of forensic medicine.

Concerning the several other tests that have been suggested for the purpose of ascertaining whether a child is born dead or alive, we shall only mention, and this very briefly, those of Ploucquet and Daniel. It is well known that the lungs previous to respiration receive their supply of blood entirely from the bronchial arteries, none passing into them from the pulmonary arteries. As soon, however, as this process becomes established, the ramifications of the pulmonary arteries are filled with blood, and in consequence of this the real weight of the lungs is considerably increased. It was on this difference, between the weight of the pulmonary organs before and after natural breathing, that Ploucquet founded his test for producing additional evidence respecting the detection of respiration in cases of infanticide. He found, on examination, that the lungs of a still-born child were to the rest of the body as 1 to 70; whereas those of a child that had arrived at the full period, and which had respired, were in the proportion of

2 to 70, or as 1 to 35. This test, an account of which was first published in 1777, and which is sometimes called the *static test*, or, after its inventor, the *test of Ploucquet*, has been found highly objectionable—in fact, perfectly useless—on the ground that it is utterly impossible to establish any standard in relation to the weight of the lungs before and after respiration, for practical purposes. As much may be said in respect to the test of Daniel, who stated that we might judge of the existence of respiration by the increase of weight given to a certain quantity of water, in which the lungs had been strongly compressed, on the self evident basis that this fluid would thus gain precisely what the pulmonary organs would lose by the expulsion of their contents.

Another test proposed by Daniel, and which is no better than the one just referred to, is founded on the arched appearance and augmented size of the chest. In a child that has not breathed, the thorax is flat and compressed; in one that has, it is vaulted and expanded. Now, by taking the dimensions of this part of the body in an infant that is still-born, and comparing them with one that has respired, Daniel thought that it was possible to judge as to the reality of breathing. Unfortunately for this test, all children are not of the same size, some being large and others small about the chest, and hence it must of course be discarded as useless, utterly unfit for the purpose for which it was suggested.

We must now bring our remarks to a close, although our notice scarcely extends through more than about two thirds of the first volume. We had intended making some remarks on several of the remaining chapters, but our review is already so "elongated" that we are necessarily obliged to stop. At some future period, we shall probably take up the second volume, especially the chapter on medical evidence, and endeavor to lay before our readers some of its valuable contents. In the meantime, we beg leave to recommend the work to the notice of our medical brethren throughout the Valley of the Mississippi, as being decidedly the most *learned, lucid, and comprehensive* treatise on the subject of Medical

Jurisprudence in any language; a treatise which we do not hesitate to say, does honor alike to our country and to the age in which we live, and which is destined to confer lasting credit on its authors, especially on him who projected it, and who has thus become, as it were, the pioneer in this branch of science in the United States of America.

Some of our readers may, perhaps, ask the question, where the necessity of so long a review of a work that has already been so many years before the profession? We answer, because the present edition, although it retains many of the most prominent features of the first, is yet so entirely different, from the numerous and important additions made by the authors, that it may, in truth, be said to be a new work. Every chapter has been carefully revised, several, indeed, have been nearly rewritten, and two, entirely new, have been added. These facts, therefore, must plead our apology, if apology be necessary. In conclusion, we cannot lay aside our pen without again expressing the hope that every physician in the West, who has at heart the cause of science, of humanity, and of self interest, will procure a copy of this able work, without which his library, however rich in other respects, will be entirely incomplete. S. D. G.

